# Seroprevalence and risk factors associated with bovine herpesvirus 1 in dairy herds of Colombia

**DOI:** 10.14202/vetworld.2022.1550-1556

**Published:** 2022-06-27

**Authors:** Aura Daniela Ortiz-González, H. Alexander Lopez Buitrago, Diana María Bulla-Castañeda, D. Johana Lancheros-Buitrago, Diego Jose Garcia-Corredor, Adriana Maria Díaz-Anaya, Julio Cesar Tobón-Torreglosa, Diego Ortiz-Ortega, Martín Orlando Pulido-Medellín

**Affiliations:** 1 Grupo de Investigación en Medicina Veterinaria y Zootecnia, Universidad Pedagógica y Tecnológica de Colombia, Tunja, Colombia; 2 Doctoral Program in Biomedical and Pharmaceutical Sciences, University of Namur, Namur, Belgium; 3 Compañía Colombiana de Productos Veterinarios, Bogotá, Colombia; 4 Corporación Colombiana de Investigación Agropecuaria, Mosquera, Colombia

**Keywords:** cattle, cattle diseases, enzyme-linked immunosorbent assay, Infectious bovine rhinotracheitis

## Abstract

**Background and Aim:**

Infectious bovine rhinotracheitis (IBR) is an infectious disease widely distributed globally and is considered the main cause of various reproductive and respiratory tract diseases in cattle and buffaloes. This study aimed to estimate seroprevalence and determine risk factors associated with the presentation of IBR in the municipality of Sotaquirá, Boyacá (Colombia).

**Materials and Methods:**

A descriptive cross-sectional study with simple random sampling was performed, and the sample size was 1,000 cattle. Blood samples were obtained by coccygeal venipuncture and processed through indirect enzyme-linked immunosorbent assay using the Synbiotics^®^ kit (Zoetis, New Jersey, USA) with a sensitivity and specificity of 96% and 98%, respectively. Data were processed using the statistical program EpiInfo^®^ (Centers for Disease Control and Prevention; Atlanta, Georgia).

**Results:**

A high seroprevalence of 57.5% was established. Seroprevalence was the highest in cattle >4 years of age (65.0% apparent seroprevalence [AS]; 67% true seroprevalence [TS]) and in the Holstein breed (65.5% AS; 67.8% TS). The breed and age of the animals were significantly associated with each other. The Holstein breed, age group >4 years, uncertified semen, and fetal death were established as risk factors for IBR. In comparison, the age groups of <1 and 1–2 years and the Normande breed were established as protective factors against the bovine herpesvirus-1 virus.

**Conclusion:**

Management factors, such as livestock from other owners and animal purchases, which affect disease presentation, are evident. The implementation and development of novel prevention and control measures for IBR at the national level are necessary.

## Introduction

Infectious bovine rhinotracheitis (IBR) is an infectious and contagious disease that is widely distributed globally, with an obligatory declaration by the World Organization for Animal Health when disease outbreaks occur in IBR-free member states or IBR-free zones of European Union countries due to its importance in terms of public health and international trade of animals and animal products [1]. IBR is caused by bovine herpesvirus 1 (BHV-1), classified within the Herpesviridae family [2], which is the main cause of several respiratory and reproductive tract diseases in cattle and buffaloes [3, 4]. BHV-1 has a wide host range, which considerably affects animal trade and causes losses of > 3 billion dollars annually in the global livestock industry [5]. Recently, two subtypes of BHV-1 have been described. Subtype 1 includes strains that cause respiratory diseases, such as IBR, essentially characterized by the occurrence of exudative rhinotracheitis that affects the major bronchi of infected animals [6]. Subtype 2 includes strains that cause genital diseases such as infectious pustular vulvovaginitis and infectious balanoposthitis [7].

Virus transmission can be indirect through contaminated people, equipment, semen (by natural mating or artificial insemination), and even through embryo transfer. Direct transmission can occur through aerosols or contact with respiratory, ocular, or reproductive tract secretions of infected animals, thereby facilitating its dissemination in livestock herds [8]. Infected animals remain carriers for the remainder of their lives. When cattle with latent BHV-1 infection are exposed to natural stressors, such as movement, extreme weather conditions, overcrowding, or immunosuppressive treatments (e.g., dexamethasone), reactivation of the latent virus occurs, leading to its transmission to susceptible hosts [9]. It is an acute disease characterized by general depression, respiratory signs, fever up to 42°C, and inappetence, resulting in a decrease in weight and milk production [3]. Likewise, on certain occasions, this virus is associated with the presentation of mastitis, metritis, abortions, infertility, altered estrous cycles, and even epididymitis in males affected by the disease [10].

BHV-1 can produce systemic and neurological diseases because it establishes an underlying infection in cattle, principally in sensorial neurons in the trigeminal ganglia or dorsal root ganglia [9]. Besides affecting the health and welfare of animals, it causes considerable losses to production caused by increased indirect costs associated with veterinary diagnosis, decreased dairy production, weight loss, and death [4]. In Boyacá, few investigations have been conducted regarding this disease [11, 12].

Therefore, this study aimed to estimate the seroprevalence and determine risk factors associated with BHV-1 seropositivity in Sotaquirá, Boyacá.

## Materials and Methods

### Ethical approval and informed consent

The study was approved by the Ethic Committee of Universidad Pedagógica y Tecnológica de Colombia. This study was performed in compliance with Law 576 of the 2000 Code of Ethics in Veterinary Medicine and Law 84 of the 1989 Statue of Animal Protection of the Republic of Colombia. Written informed consent was obtained from cattle owners before sample collection for this study.

### Study period and location

The study was carried out from July 2018 to July 2019. The municipality of Sotaquirá is located in the Central Province of the department of Boyacá. It has a 288.65 km^2^ area, an altitude of 2681 m above sea level. The average temperature is 14°C, and the topography varies between valleys and mountains. Its primary economic activity is based on agriculture and livestock. This municipality has stood out for being part of the dairy corridor of the Boyacá department due to its high milk productivity [13].

### Sample size

A descriptive (cross-sectional) cutoff type study was developed through simple random sampling and proportional distribution to the sample size, which was calculated by considering the bovine population of Sotaquirá with 19,333 heads of cattle [14] and implementing the following equation:


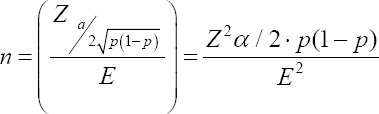


Where: n = sample size; E = accepted error; p = expected value of the proportion; a = tail probability. The sample obtained was 1000 individuals with a sampling fraction of 5.17%. Likewise, a margin error of 3.1% was considered, with a confidence level of 95% and an expected prevalence of 50%, considering no studies of this type have been conducted in the area.

### Variables

An epidemiological survey was designed and completed in each cattle herd, including information that allowed the variables to be classified into two categories. The variables associated with the animal taken into consideration were sex, breed, age, and reproductive variables that were associated with BHV-1 as per the literature. In contrast, herd variables, including management practices, were evaluated to establish the main risk factors associated with disease presentation.

### Sample collection and processing

A total of 1000 blood samples from cattle were collected via coccygeal venipuncture from healthy females and males of different age groups. Animals were classified according to Ayrshire, Holstein, Jersey, Normande, Zebu, and Crossbreeds, distributed in 65 small and medium herds without vaccination history. The samples were deposited in Vacutainer® vacuum tubes without anticoagulants (Becton, Dickinson and Company, USA). These samples were appropriately labeled, refrigerated (4°C), transferred to the Veterinary Parasitology Laboratory of the Universidad Pedagógica y Tecnológica de Colombia, and centrifuged at 1,327.6× g for 10 min. The resulting blood sera were deposited in Eppendorf tubes for subsequent storage at −20°C [15]. The samples were processed via indirect enzyme-linked immunosorbent assay (ELISA) using the Synbiotics^®^ commercial kit (sensitivity - 96%; and specificity - 98%, Zoetis, USA) according to the manufacturer’s instructions.

### Statistical analysis

Data were sorted, consolidated, and processed using the statistical program EpiInfo^®^ (Centers for Disease Control and Prevention; Atlanta, Georgia). The determining factors were established by calculating the prevalence ratio (PR). The dependent variable (Y) included the obtained serological results, and the independent variables (X) were all the determining factors recognized in the structured epidemiological survey applied during the sample collection. Once these factors were established, a stratified logistic regression was performed to test for confounding and identify the simultaneous interaction between the variables significantly associated with BHV-1 [16].

## Results

Out of 1000 cattle, 869 were females and 131 were males. The apparent seroprevalence (AS) was 57.5%; 516 females (59.4%) and 59 males (45.0%) were positive for antibodies against the virus (Table-1). The true seroprevalence was 59% by considering the AS, sensitivity (96%), and specificity (98%) of the ELISA kit.

**Table 1:** AP and PR of IBR by bovine sex and breed in cattle from Sotaquirá, Boyacá.

Category	n	Positives	AS (%)	TS (%)
Sex				
Female	869	516	59.4	61.1
Male	131	59	45	45.7
Breed				
Holstein	601	395	65.7	67.8
Ayrshire	11	7	63.6	65.5
Crossbreed	88	46	52.3	53.5
Cebu	22	11	50	51.1
Jersey	21	10	47.6	48.5
Normando	257	106	41.2	41.7

AS=Apparent seroprevalence, TS=True seroprevalence, IBR=Infectious bovine rhinotracheitis

Among the breeds evaluated in the study, Holstein (65.5%) and Ayrshire (63.6%) had the highest seroprevalence, followed by Crossbreds (52.3%), Zebu (50.0%), and Jersey (47.6%). The Normande breed had the lowest seroprevalence (41.2%; Table-1). In addition, cattle > 4 years old had the highest seroprevalence (65.0%), followed by cattle < 1 year old (55.9%) and 1–2 years old (48.8%); and cattle 2–4 years old (42.9%) had the lowest seroprevalence (Table-2).

**Table 2:** AP and PR of IBR by age groups in cattle from Sotaquirá, Boyacá.

Age	n	Positives	AS (%)	TS (%)
< 1 year (6–12 months)	179	100	55.9	57.3
1–2 years	209	102	48.8	49.8
2–4 years	112	48	42.9	43.5
> 4 years	500	325	65	67

AS=Apparent seroprevalence, TS=True seroprevalence, IBR=Infectious bovine rhinotracheitis

Cattle belonging to the Holstein (p = 0.000) and Normande (p = 0.000) breeds had a significant statistical association with the presence of antibodies against BHV-1. Similarly, cattle of age groups < 1 year (p = 0.000), 1–2 years (p = 0.002), and >4 years (p = 0.000001) showed a significant statistical relationship presence of antibodies against IBR (p ≤ 0.05), and bovine sex did not show an association (p < 0.05), indicating that the antibodies against BHV-1 is associated with the breed and age of the cattle, while bovine sex was not significantly associated with the presence of antibodies against IBR (Table-3).

**Table 3:** Analysis of breed, sex and group age as possible risk factors associated with IBR infections. Results are shown as PR and 95% CI.

Variable	Category	PR	95% CI	p-value
Breed	Holstein	1.6013	1.3893–1.8457	**0.000**
	Normando	0.6277	0.5462–0.7213	**0.000**
	Ayrshire	1.1706	0.5339–2.5668	0.46375049
	Zebu	0.8466	0.5539–1.294	0.30571159
	Crossbreed	0.8799	0.698–1.1092	0.17712248
	Jersey	0.8073	0.5335–1.2217	0.23985024
Sex	Female	0.9756	0.7134–1.0855	0.073357321
	Male	1.1532	0.958–1.3636	0.069397203
Age group	< 1 year	1.4286	1.2318–1.6567	**0.00000105**
	1–2 years	0.7853	0.671–0.919	**0.00279997**
	2–4 years	1.0699	0.8864–1.2914	0.26559333
	> 4 years	1.5286	1.1318–1.7567	**0.000001**

IBR=Infectious bovine rhinotracheitis, PR=Prevalence ratio, CI=Confidence interval. Significance is denoted by p < 0.05; The numbers in bold denotes the statistical association with the significance

Among the management practices implemented on farms, the presence of damaged fences (p = 0.009234232) at farm boundaries was significantly associated with IBR seropositivity, indicating that disease manifestation is associated with management practices (Table-4).

**Table 4:** Analysis of management practices as possible risk factors associated with IBR infections. Results are shown as PR and 95% CI.

Variable	PR	95% CI	p-value
Corral	1.108	0.9554–1.2851	0.101493787
Vaccination	1.6083	0.3046–1.2148	0.078863099
Damaged fences	1.4435	1.2531–1.6629	**0.009234232**

IBR=Infectious bovine rhinotracheitis, PR=Prevalence ratio, CI=Confidence interval. Significance is denoted by p < 0.05; The numbers in bold denotes the statistical association with the significance

In contrast, uncertified semen (p = 0.000021), abortion (p = 0.005890), and fetal death (p = 0.0000) were significantly associated with antibodies against BHV-1 in the cattle (Table-5).

**Table 5:** Analysis of reproductive variables as possible risk factors associated with IBR infections. Results are shown as PR and 95% CI.

Variable	PR	95% CI	p-value
Uncertified semen	1.9205	1.3327–1.7502	**0.000021**
Abortion	1.2094	1.0464–1.3979	**0.005890**
Fetal death	2.5517	1.885–3.4533	**0.0000**

IBR=Infectious bovine rhinotracheitis, PR=Prevalence ratio, CI=Confidence interval. Significance is denoted by p < 0.05; The numbers in bold denotes the statistical association with the significance

The PR and confidence interval (CI) values allowed the identification of variables associated with cattle that contributed as risk factors, including the Holstein (1.6013), Zebu (0.8466 CI = 0.5539–1.294), Crossbred (0.8799 CI = 0.698–1.1092), Jersey (0.8073 CI = 0.5335–1.2217), and individuals < 1 year (1.4286) and > 4 years (1.5286). Based on the reproductive variables, the implementation of noncertified semen (1.9205), abortion (1.2094), and fetal death (2.5517) presented the same condition. Based on the management practices on farms, damaged fences were established as a risk factor (1.4435). However, logistic regression corroborated that the Holstein breed, age groups < 1 and > 4 years, presence of damaged fences, uncertified semen, and fetal death were the risk factors of IBR (Table-6).

**Table 6:** Analysis of variables as possible risk factors associated with IBR infections.

Variable	Odds ratio	LCI (95%)	SCI (95%)	p-value
Holstein	0.3329	1.8	3.0236	**0.000**
Zebu	0.7340	0.3152	1.7094	0.4734
Crossbreed	0.7930	0.5115	1.2294	0.2998
Jersey	0.6661	0.2803	1.5832	0.3577
< 1 year	0.4101	0.307	0.5479	**0.000**
> 4 years	1.8571	1.4406	2.3941	**0.000**
Damaged fences	0.7107	0.5494	0.9193	**0.0093**
Uncertified semen	2.7918	1.1251	6.9277	**0.0268**
Abortion	1.5444	0.5489	4.3449	0.4102
Fetal death	3.2093	1.7598	5.8529	**0.0001**

IBR=Infectious bovine rhinotracheitis, SCI=Superior confidence interval; LCI=Lower confidence interval. Significance is denoted by a p < 0.05; p < 0.05; The numbers in bold denotes the statistical association with the significance

## Discussion

Seroprevalence and risk factors associated with the presentation of antibodies against the virus that causes IBR were determined. The seroprevalence noted in the municipality of Sotaquirá was 57.5%, a percentage higher than those reported in Toca (65.5%) and Montería (Colombia; 74.7%) [12, 17]. However, this was a low prevalence compared with that in Caquetá (90% [18] and 73.13% [19]) and Antioquia (85.51% [20]. The variation among the studied regions may be a result of animal density, herd size, and sanitary and reproductive management [21].

Prevalence in Brazil (71.30%, [10]), Peru (67.00% [22]), Spain (99.92% [23]), and Mexico (76.32% [24]) have been reported, indicating a wide distribution of the virus. The presented variations were due to the fact that factors such as poor sanitary management, lack of vaccination, and infected animals’ introduction to the herds, directly affected the virus. In addition, the acquisition of stallions without sanitary control contributes to aggravating the animal health status of herds because this disease is transmitted through the venereal route [10].

Holstein and Ayrshire had the highest seroprevalences among the evaluated breeds; this finding is similar to that reported by Ortíz-González *et al*. [12]. However, Sibhat *et al*. [25] have reported that the Jersey breed and crosses of Holstein–Friesian by Zebu have the highest seroprevalences of 62% and 41.8%, respectively. Holstein and Normande breeds were significantly associated (p ≤ 0.05) with the presence of antibodies against IBR; this finding is in contrast with the findings of Ortíz-González *et al*. [12] and Sibhat *et al*. [25]. Likewise, the Normande breed was 0.6277 (PR) less likely to have BHV-1 antibodies, and the Holstein breed was determined to be a risk factor for IBR infection. However, cattle of all breeds are susceptible to this disease [26]. In addition, factors such as acquiring infected cattle, participation in agricultural shows, and increasing the herd size and production system type, may influence the virus seropositivity [27, 28].

Individuals > 4 years had the highest seroprevalence, which is in agreement with the findings of Ochoa *et al*. [11], Santman-Berends *et al*. [29], and Sibhat *et al*. [25], who reported that the highest seroprevalences were in older age groups. Animals over the age of 24 months have a higher risk of infection; at this age, cows attain reproductive maturity through artificial insemination or direct mating, events classified as risk factors for virus transmission [11].

A significant statistical association was established between virus seropositivity and age groups, which is in agreement with the findings of Sibhat *et al*. [25] and Serem *et al*. [30]. However, this result differed from that of Segura-Correa *et al*. [31], who reported no significant statistical association between virus seropositivity and cattle age. Moreover, the age group > 4 years is a risk factor for the presentation of IBR. Possibly after the decrease in maternal immunity, the incidence of seroconversion increases with age [32] and attains the highest rate in the first 24 months [32, 33].

However, because adult animals have generally already acquired immunity following natural infections previously, the seroconversion rate decreases caused by herd immunity [32, 33]. Therefore, higher seroprevalence in adult animals in cross-sectional studies could be the result of an accumulation of seropositivity over an extended period and may not necessarily be an indication of age-dependent susceptibility [25].

A higher IBR seroprevalence was found in females than in males, in agreement with the findings of Derrar *et al*. [34], who reported the presence of antibodies in 32.17% of females and 19.91% of males. This could be because more females than males were sampled. Likewise, no association was reported with the sex of the animals, which did not agree with the findings of Muñoz *et al*. [19] but agreed with those of Derrar *et al*. [34], who reported the absence of a relationship between the disease and sex, thereby indicating that virus presentation is independent of the sex of the cattle.

A significant statistical association was established between the presence of damaged fences and virus seropositivity. This variable was also determined as a risk factor for the disease, indicating that disease manifestation is associated with management practices conducted in each farm, in agreement with the findings reported by Kaddour *et al*. [35], who indicated that the lack of specific infrastructure for cattle is a risk factor associated with BHV-1 infection. Likewise, Segura-Correa *et al*. [31] and Serem *et al*. [30] identified that the presence of animals from other farms directly influences disease presentation and the presence of other animals, such as goats and their offspring. Thus, the uncontrolled movement of visitors and livestock to the farm, unreliable records of vaccination dates, lack of vehicle disinfection posts, and absence of quarantine and control tests of acquired animals are influential factors associated with disease transmission [26, 36].

In Sotaquirá, a significant statistical association was found with the presentation of abortions in herds, which is in agreement with the findings of Mahajan *et al*. [37]. A statistical association between the presence of abortion and the disease was reported but differed from the observation made by Ochoa *et al*. [11] and Ortíz-González *et al*. [12], who did not report this relationship with abortion. In addition, gestation in virus-infected cattle can be carried to term with the birth of dead calves or calves that died shortly after birth or within the first 2 weeks [38].

Fetal death was established as a risk factor associated with the presentation of IBR. This may occur because, after viremia, the virus crosses the placenta–blood barrier to lethally infect the fetus, causing death and subsequent expulsion. Although the route of the virus from the placenta to the fetus is unknown, viral lesions regularly observed in the fetal liver suggest that the hematogenous dissemination occurs through the umbilical vein [26]. Likewise, the proportion of animals with uterine infection and fetal membrane retention was frequent in cattle seropositive to BHV-1 exposure, associating the presence of the disease more frequently with dairy cows that had abortions and fetal death history compared with cows that did not [39].

Finally, a study has reported infected sires to be a transmission factor because BHV-1 is transmissible through the venereal route [20]. Consequently, the use of uncertified semen has been reported as a risk factor. In addition, infected bulls tend to be carriers and could eliminate the virus for > 1 year despite negative serological tests. On the other hand, the semen sample presents some particularly cytotoxic factors that avoid to verify the Pathogen’s presence. Therefore, evaluation and assessment of the semens obtained from stallions and their clinical history are indispensable [40].

## Conclusion

Serological findings of IBR in Sotaquirá demonstrated the presence of the disease in the area. This can harm many livestock farms due to an increase in production costs associated with veterinary diagnosis, treatment of clinical manifestations, and death of virus-carrying animals. Moreover, a significant relationship between the presence of reproductive alterations and the disease was evidenced, indicating that this virus can affect cattle and cause abortions and fetal death in these animals. Similarly, other management factors influence the presentation and transmission of the disease within the herd such as the presence of cattle from different herds, acquisition of animals, and use of uncertified semen. Consequently, it is essential to implement and develop new measures against IBR at the national level to establish plans for the prevention and control of the pathology.

## Authors’ Contributions

MOPM and DOO: performed the study. DMB, ADO, HALB, DJG, and DO: Performed formal analysis and data curation. ADO, DMB, AMD, DJL, DJG, and MOP: Wrote original draft and reviewed the final version of the manuscript. JCT, DO, DJL, and MOP: Reviewed the final version of the manuscript. DO: Designed the study and collected the data. All authors have read and approved the final manuscript.
